# The Photocycle of Bacteriophytochrome Is Initiated by Counterclockwise Chromophore Isomerization

**DOI:** 10.1021/acs.jpclett.2c00899

**Published:** 2022-05-16

**Authors:** Dmitry Morozov, Vaibhav Modi, Vladimir Mironov, Gerrit Groenhof

**Affiliations:** † Nanoscience Center and Department of Chemistry, University of Jyväskylä, P.O. Box 35, 40014 Jyväskylä, Finland; ‡ Department of Chemistry, Kyungpook National University, Daegu 702-701, South Korea

## Abstract

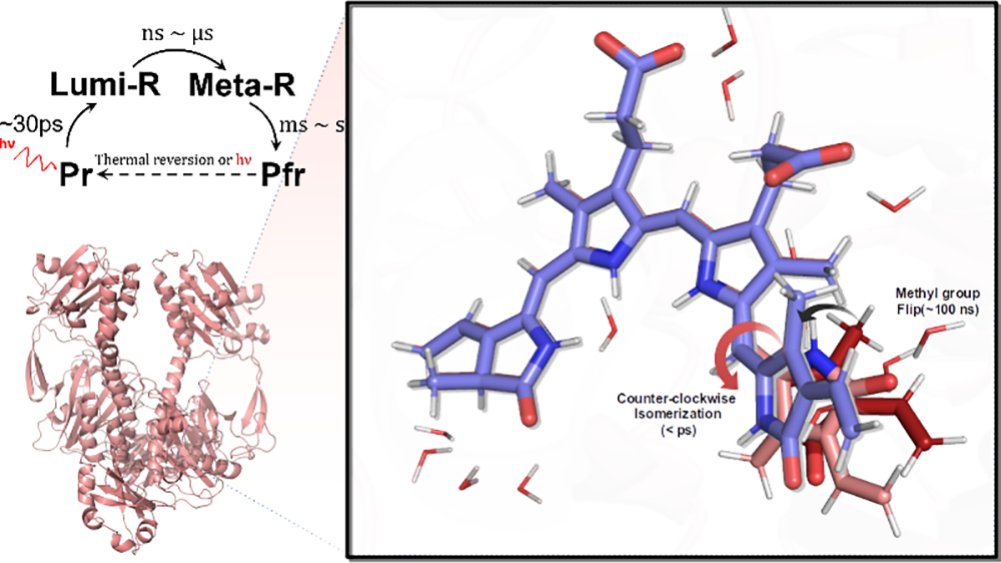

Photoactivation of bacteriophytochrome involves a cis–trans photoisomerization of a biliverdin chromophore, but neither the precise sequence of events nor the direction of the isomerization is known. Here, we used nonadiabatic molecular dynamics simulations on the photosensory protein dimer to resolve the isomerization mechanism in atomic detail. In our simulations the photoisomerization of the D ring occurs in the counterclockwise direction. On a subpicosecond time scale, the photoexcited chromophore adopts a short-lived intermediate with a highly twisted configuration stabilized by an extended hydrogen-bonding network. Within tens of picoseconds, these hydrogen bonds break, allowing the chromophore to adopt a more planar configuration, which we assign to the early Lumi-R state. The isomerization process is completed via helix inversion of the biliverdin chromophore to form the late Lumi-R state. The mechanistic insights into the photoisomerization process are essential to understand how bacteriophytochrome has evolved to mediate photoactivation and to engineer this protein for new applications.

Phytochrome is a photoreceptor protein in plants, fungi, and bacteria that mediates the response of these organisms to red and far-red light.^1−8^ Upon photoactivation, the protein dimer interconverts reversibly between a red (P_r_) and a far-red (P_fr_) absorbing state.^7,9^ Time-resolved wide-angle X-ray scattering of the photosensory domain suggests significant structural changes between these states^10^ that control the activity of a histidine kinase (HK) domain.^11−13^ Based on X-ray structures of the P_r_ and P_fr_ conformations of the photosensory unit (i.e., the protein without the HK domain),^10,14^ the first step in this signal transduction pathway is assumed to be the photoisomerization of a covalently bound tetrapyrrole biliverdin chromophore (Figure 1a), but the exact mechanism is not known. On the basis of changes in circular dichroism between the P_r_ and P_fr_ states of phytochromes from various organisms, Lagarias and co-workers proposed that the isomerization proceeds counterclockwise in the *phytobilin* phytochromes of plants and cyanobacteria but clockwise in the *biliverdin* phytochrome of bacteria and fungi.^15^ In contrast, recent time-resolved serial femtosecond X-ray diffraction (trSFX) experiments on the chromophore binding domain (CBD) of the *Deinococcus radiodurans* phytochrome (DrBphP) suggest a counterclockwise photoisomerization of the biliverdin chromophore binding phytochromes.^16^

**Figure 1:**
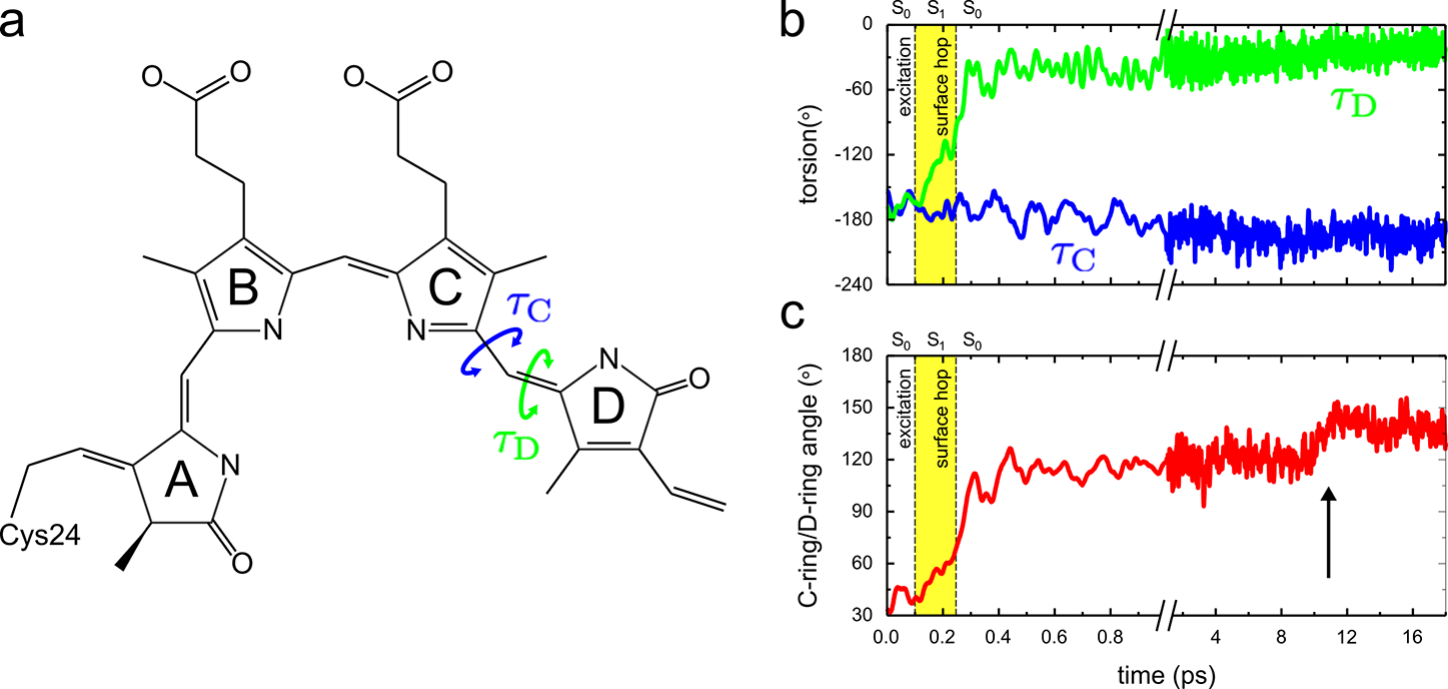
(a) Schematic representation of the biliverdin chromophore in DrBphP. The rings are labeled A, B, C, and D. The torsion angles for τ_C_ and τ_D_ are highlighted. (b) Time evolution of torsions τ_C_ and τ_D_. Note the change in the scale on the time axis at 1 ps. The yellow background indicates that the system is in the electronic excited state (S_1_). (c) Angle between the normals of the C and D rings. The vertical arrow indicates a structural relaxation of the chromophore (see Figure 3).

In the trSFX experiments on the CBD of DrBphP two snapshots of what is essentially a dynamic process that spans multiple time scales, were captured at pump–probe delays of 1 and 10 ps.^16^ The pump laser, with which the photoisomerization was initiated, had high power, and some of the structural changes may therefore have been induced by multiphoton absorption. To investigate the photoisomerization mechanism in the single-photon excitation regime, we resort to nonadiabatic molecular dynamics simulations. While the accuracy of atomistic computer simulations remains a matter of concern despite the tremendous progress in hardware and software, we note that our previous simulations correctly predicted the sequence of events in the photoisomerization process in a related protein.^17,18^

Here, we used the same hybrid quantum mechanics/molecular mechanics (QM/MM) approach^19,20^ to follow the photoinduced dynamics in the complete photosensory dimer (CBD-PHY) of DrBphP.^10^ In our QM/MM model,^21^ one biliverdin chromophore was described at the SA2-CASSCF(6,6)/3-21G level of theory,^22^ while the rest of the system, including the rest of the monomer as well as the complete other monomer, waters, and ions were treated with the Amber03 force field.^23^ All details of the nonadiabatic simulations are provided as Supporting Information, including a validation of our model at the correlated xMCQDPT2/SA3-CASSCF(12,12)/cc-pVDZ level of theory.^24^

Immediately after resonant photoexcitation to the excited state (S_1_) potential energy surface, the chromophore relaxes on a subpicosecond time scale from the Franck–Condon region to the conical intersection seam between the ground state (S_0_) and excited state in 33 out of 50 simulations (Table S1, Supporting Information). Upon reaching the S_1_/S_0_ conical intersection, the system decays to the ground state. In four trajectories, the chromophore reaches a new configuration (discussed below), while in the other 29 trajectories, the chromophore rapidly relaxes back into the original ZZZssa geometry, in line with the very low quantum yield of photoactivation in bacterial phytochromes.^25−27^ In 17 out of 50 simulations, the chromophore remains planar and does not decay to S_0_ on the 5 ps time scale of the simulation. Although 5 ps is orders of magnitude shorter than the measured excited-state lifetime of CBD-PHY (170 ps),^28^ we speculate nevertheless that these trajectories represent longer-lived substates in the protein conformational ensemble that are responsible for fluorescence.

In Figure 1, we show the evolution of the angle between the normal of the C ring and the normal of the D ring as well as of the τ_C_ and τ_D_ torsions in one of the trajectories that forms a photoproduct. In the 33 trajectories that reach the conical intersection, the initial relaxation process is highly similar and proceeds via twisting the τ_D_ torsion angle to about 90° (Figures 1b and 2b). Along this reaction coordinate, the gap between the S_0_ and S_1_ states decreases until it disappears at the S_1_/S_0_ intersection (Figures S3 and S4), where a diabatic surface hop takes the system back to the electronic ground state. The excited-state decay process in these 33 trajectories takes less than a picosecond on average (Table S1), which is in line with recent simulations of the CBD monomer,^29^ but seems to contradict the 170 ps excited-state lifetime measured experimentally for this system.^28^ We note, however, that the excited-state decay in phytochromes is a highly heterogeneous process and that fits to the excited-state lifetime in pump–probe experiments require multiple components,^27,28,30−36^ including an ultrafast subpicosecond component.^25,28,37^ We therefore tentatively assign this subpicosecond component to the ultrafast photoinduced rotation of the D ring and attribute the slower components to the protein conformations, in which the chromophore remains planar without deactivating (Table S1).

**Figure 2:**
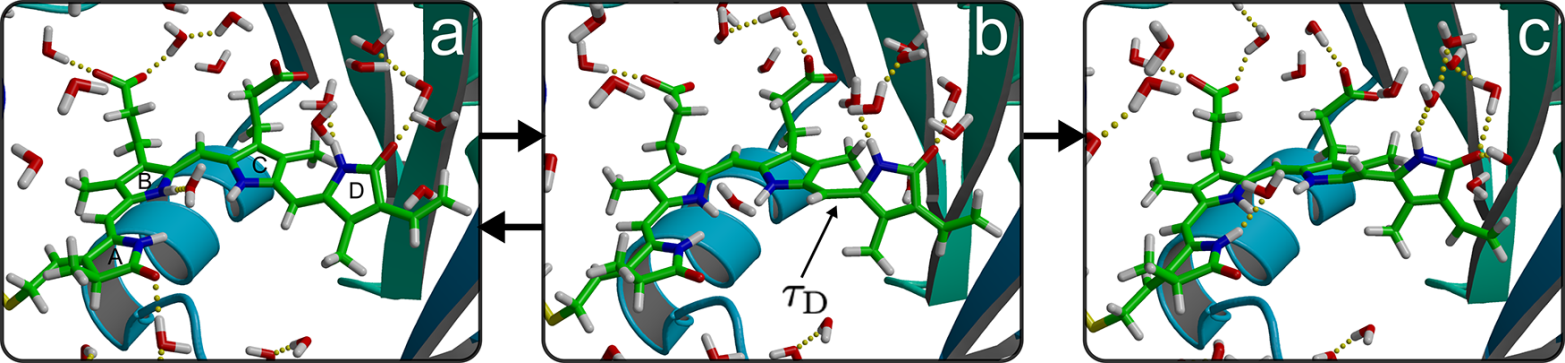
(a) Snapshot at the start of a simulation. The chromophore is in the ZZZssa configuration. (b) Snapshot when the trajectory reaches the S_1_/S_0_ conical intersection hyperline. The τ_D_ torsion (arrow) is around 90° (Figure 1b). (c) Snapshot at 1 ps after excited-state decay through the conical intersection. Both τ_C_ and τ_D_ are close to their equilibrium values, but the D ring is still twisted with respect to the C ring and the rest of the chromophore (Figure 1c). This strained configuration is stabilized by a hydrogen bond network between the D ring nitrogen and carboxyl oxygen atoms, on the one hand, and buried water molecules, on the other hand.

After the radiationless decay from the excited state, the τ_D_ torsion angle reverts back in 29 trajectories, restoring the ZZZssa configuration of the chromophore. In the other four trajectories, the τ_D_ torsion angle rotates further on the ground state to reach a ZZEssa configuration (Figure 2b). This configuration is, however, strained, as indicated by an almost perpendicular orientation of the D ring with respect to the rest of the chromophore in Figure 2c. In this configuration, which we term I_0_, the angle between the C and D rings is around 120° and remains at that angle for at least 10 ps (Figure 1c). As shown in Figure 2c, a hydrogen-bonding network that involves multiple buried water molecules stabilizes the D ring in this orientation, with the amino (N–H) and carboxyl (C=O) groups acting as donor and acceptor, respectively. The formation of this twisted configuration on a subpicosecond time scale is supported both by time-resolved X-ray crystallography, which resolved such twisted structure on similar time scales,^16^ and by femtosecond stimulated Raman spectroscopy,^37^ which probed the rise of signals associated with out-of-plane distortions, with a 450 fs time constant. A comparison between the twisted intermediate found in our simulations and the 1 ps structure refined by Claesson et al.^16^ in Figure S10 reveals that the simulations predict a very similar chromophore configuration but not the large displacement of the pyrrole water molecule. We speculate therefore that the photodissociation of the pyrrole water observed in trSFX might have been induced by a multiphoton absorption process due to the very high laser power in the experiments.

Eventually, the hydrogen-bonding network breaks up in the QM/MM simulations, and the chromophore relaxes into a more planar configuration, as shown by the transition around 11 ps in Figure 1c. In this configuration, which is stable throughout the rest of the simulation, the chromophore is further stabilized by a new hydrogen bond between the D ring amino group (N–H) and the hydroxyl group of the conserved Tyr263 (Figure 3). Because mutating this residue into a phenylalanine hinders the formation of Lumi-R,^39^ we attribute the configuration in Figure 3b to the early Lumi-R state, which is also observed on similar time scales in transient absorption spectroscopy experiments.^28^ Statistically, the number of trajectories is small but nevertheless yields a consistent picture of the photoisomerization mechanism.

**Figure 3:**
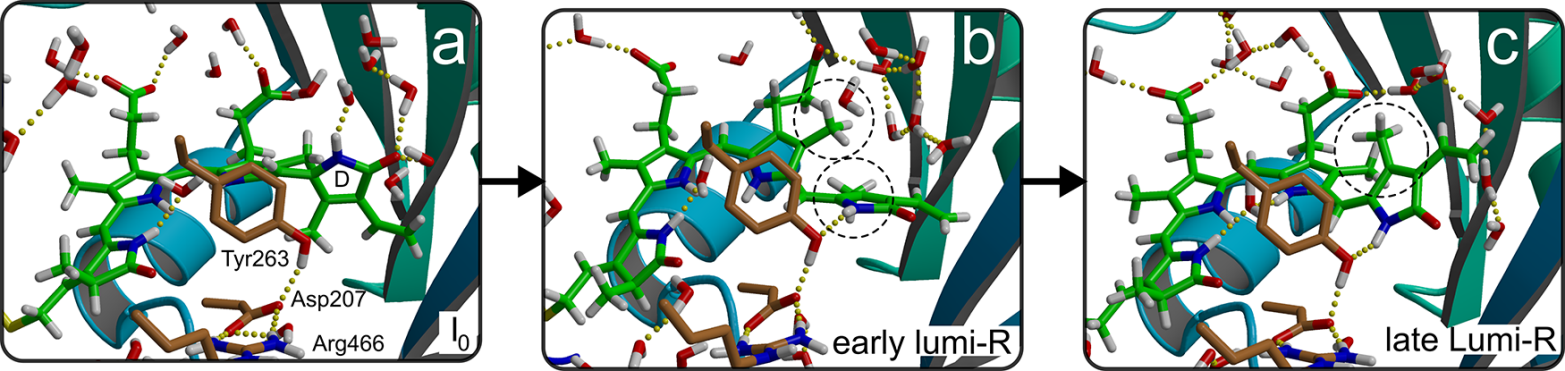
(a) Configuration in the first local minimum on S_0_ after decay from S_1_ (I_0_, same as Figure 2c but with additional structural details). The chromophore is twisted with the D ring almost perpendicular to the C ring and the rest of the chromophore. The D ring forms an extensive hydrogen-bonding network involving multiple buried water molecules. (b) Configuration after 15 ps. The D ring is more planar with respect to the rest of the chromophore and donates a hydrogen bond to the hydroxyl group of Tyr263. The latter configuration is further stabilized by a hydrogen-bonding network involving Asp207 and Arg466. We assign this configuration to the early Lumi-R state in the photocycle. The circles emphasize the relative positions of the methyl substituents of the C and D rings. (c) Configuration after flipping the methyl groups of the C and D ring in umbrella sampling simulations (see the Supporting Information for details). We assign this configuration, in which the D ring has undergone a 180° rotation with respect to the P_r_ resting state, to the late Lumi-R state.^38^

Although the early Lumi-R intermediate is stable in the rest of the MD simulations, the chromophore is not in the configuration observed in the X-ray structure of the activated P_fr_ state.^14^ The main difference is the facial disposition of the D ring relative to the C ring:^15^ In the P_fr_ X-ray structure the D ring adopts a β_f_ disposition, in which the methyl group of the D ring is *above* the plane of the C ring (Figure 3c),^14^ while in our early Lumi-R intermediate, the disposition of the D ring is α_f_ with its methyl substituent lying *below* the plane of the C ring (Figures 2c and 3b). To estimate the free energy barrier associated with changing the disposition of the D ring from α_f_ to β_f_ within the protein environment, we performed umbrella sampling simulations^40^ at the PBE/DZVP//Amber03 level of theory (see the Supporting Information for details). The results of these simulations, shown in Figure S8, suggest an upper bound of 33 kJ mol^–1^ for the barrier separating the α_f_ and β_f_ dispositions of the D ring. Thus, based on Eyring’s transition state theory (TST), the time scale of this inversion process would be on the order of 62 ns, which is much faster than the onset of the large protein structural changes seen in time-resolved WAXS experiments^10^ but qualitatively in line with the time scales at which a late Lumi-R state was observed in transient infrared (trIR) spectroscopy measurements.^38,41^ We therefore tentatively assign the structure in which the chromophore has already adopted the configuration of the P_fr_ state (i.e., the ZZEssa configuration with the β_f_ disposition of the D ring), while the protein is still in the P_r_ conformation, to the late Lumi-R intermediate in the photocycle (Figure 3c).

Because in a circular dichroism spectrum (CD) the Q-band absorption of the chromophore has a negative rotation in the α_f_ disposition, but a positive rotation in the β_f_ disposition,^42−44^ and the CD of this Q-band is negative in both the P_r_ and P_fr_ states, Rockwell et al. proposed a clockwise photoisomerization of the D ring.^15^ In contrast, our simulations suggest a counterclockwise rotation of the D ring, which was also observed in the simulations by Salvadori et al.^29^ To investigate the effect of the counterclockwise isomerization on the CD signal, we computed CD spectra of the P_r_ state and the structural intermediates (details in the Supporting Information). While the rotation of Q-band is negative in P_r_, late Lumi-R, and P_fr_, it is positive in the early Lumi-R intermediate (Figure S9). Thus, to verify the validity of our results, we propose to transiently probe the effects of photoabsorption on the CD spectrum with picosecond time resolution, as has been done by Mendonça and co-workers for the photoactive yellow protein.^45^

Summarizing, the results of our nonadiabatic MD simulations suggest a counterclockwise photoisomerization of the biliverdin chromophore in the phytochrome of *Deinococcus radiodurans*, which proceeds via three intermediates: I_0_, early Lumi-R, and late Lumi-R. Although the structures of these intermediates have so far not been resolved experimentally, their lifetimes are in reasonable agreement with experimental estimates.^12,37,38^ Because already in the late Lumi-R intermediate the chromophore has the same configuration as in the P_fr_ state, while the rest of the protein still adopts the P_r_ conformation, we speculate that complete chromophore isomerization is essential to trigger the conformational changes. The atomistic insights into the dynamics and interactions of the isomerization process may be useful to systematically improve phytochromes for new applications, such as optogenetics,^46,47^ or fluorescence microscopy.^47,48^

## Supporting Information Available

Complete description of the QM/MM simulations and other calculations, overviews of nonadiabatic simulations, calculated circular dichroism spectra, and comparisons of structural intermediates to time-resolved X-ray structures (PDF)
Movie S1 (MOV)
Movie S2 (MOV)
Movie S3 (MOV)
Transparent Peer Review report available (PDF)

## Supplementary Material

jz2c00899_si_001.pdf

jz2c00899_si_002.mov

jz2c00899_si_003.mov

jz2c00899_si_004.mov
