# Supplementary material for: The Photocycle of Bacteriophytochrome Is Initiated by Counterclockwise Chromophore Isomerization
Source: J Phys Chem Lett. 2022 May 16;13(20):4538–42. doi: 10.1021/acs.jpclett.2c00899 (PMC9150100; doi:10.1021/acs.jpclett.2c00899)
Supplement: Supplementary file 1 — jz2c00899_si_001.pdf [file jz2c00899_si_001.pdf]

Supporting information for
The Photocycle of Bacteriophytochrome Is Initiated by Counter-Clockwise Chromophore Isomerization

Dmitry Morozov,† Vaibhav Modi,† Vladimir Mironov,‡ and Gerrit Groenhof*,†

† *Nanoscience Center and Department of Chemistry, University of Jyväskylä, P.O. Box 35, 40014 Jyväskylä, Finland.*

‡ *Department of Chemistry, Kyungpook National University, Daegu, 702-701, South Korea.*

E-mail: gerrit.x.groenhof@jyu.fi

Contents

Simulation setup 3

Analysis of the excited state trajectories 5

Validation of chromophore QM model 5

QM/MM geometry optimization 8

QM/MM umbrella sampling simulations 9

Circular dichroism spectra of P_r, early and late Lumi-R 14

References 16

Simulation setup

All interactions were modeled with the Amber03 Molecular Mechanics (MM) force field,[1] with the parameters for the Biliverdin chromophore from Modi *et al.*[2]. The initial coordinates of the fully solvated dimeric photosensory core module of the *Deinococcus Radiodurans* phytochrome (CBD-PHY) were also taken from that work.[2] Briefly, the x-ray structure (PDB id: 4O0P) of the photosensory dimer was placed at the center of a cubic periodic simulation box ($\sim 12 \times 12 \times 12$ nm^3). Protonation states and tautomeric forms of amino acids with ionizable side chains were assigned based on pK_a estimates of H++ Server and PROPKA 3.1 at pH = 7. To the simulation box, we added 50536 TIP3P water molecules,[3] as well as 186 sodium and 151 chloride ions to neutralize the system at 0.15 M ionic strength.

The short-range attractive and repulsive dispersion interactions were described by a Lennard-Jones potential with a cut off of 1.0 nm. Electrostatic interactions were calculated at each time step using the particle mesh Ewald method[4] with a grid spacing of 0.12 nm. The LINCS algorithm was used to constrain bond lengths in the protein,[5] while SETTLE[6] was used to constrain the internal degrees of freedom of the TIP3P water molecules. With these constraints, we could propagate the classical molecular dynamics with a time step of 2 fs.

From the second half of the 100 ns MM equilibrium trajectory, 50 frames were selected at 1 ns intervals for the non-adiabatic QM/MM simulations.[7,8] In these simulations, the conjugated system of one biliverdin chromophore (Figure S1) was described at the CASSCF/3-21G level,[9] with an active space of six electrons and six orbitals that are shown in Figure S2. To validate this rather small active space and basis, we also performed calculations at the multi-reference xMCQDPT2/SA3-CASSCF(12,12)/cc-pVDZ level, as discussed below. Strict *diabatic* surface hopping was allowed only on the conical intersection hyper-line between the excited (S_1) and ground state (S_0) potential energy surfaces.[10] Although restricting hops to the S_1/S_0 seam could underestimate the population transfer,[10] we consider this an advantage if only a few trajectories can be computed. Furthermore, with this algorithm, we obtain direct information on the location of the conical intersection in our trajectories. We note that because the His260 residue, which interacts

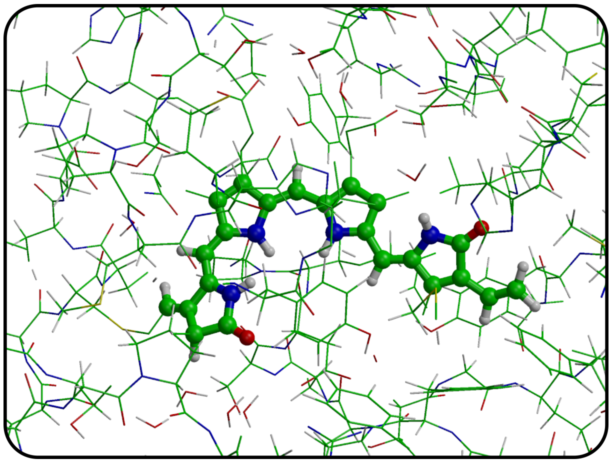

Figure S1: QM/MM model system. The QM atoms, treated at the RHF/3-21G and CIS/3-21G levels of theory for the ground (S_0) and excited states (S_1), respectively, are shown in ball-and-stick representation, while the MM atoms, modeled with the Amber03 force field[1], are shown as sticks. The hydrogen link atoms introduced along the bonds on the QM/MM interface are not shown.

with the C ring of the chromophore via π-stacking, was not included in the QM region, we could not investigate a recent hypothesis that a charge-transfer excitation between this residue and the chromophore plays a role in the photo-activation mechanism.[11] However, because these CT states have a significantly smaller oscillator strength than the main $\pi - \pi^*$ transition, we consider it a valid approximation to focus exclusively on the $\pi - \pi^*$ S_1 state in this work.

The remainder of the system, consisting of the aliphatic moieties of the chromophore, the chromophore in the second dimer, the two apo-protein monomers, water molecules and ions, were modeled with the Amber03 force field.[1] The seven bonds connecting the QM and MM subsystems (Figure S1) were replaced by constraints,[5] and the QM part was capped with hydrogen atoms. The force on these cap atoms was distributed over the two atoms of the bond. The QM system experiences the Coulomb field of all MM atoms within a 1.6 nm cut-off sphere around the QM atoms. Lennard-Jones interactions between MM and QM atoms were added. The time step in the QM/MM simulations was reduced to 1 fs. Prior to the excited state molecular dynamics simulations, the 50 snapshots were equilibrated for 1 ps at the RHF/3-21G//Amber03 QM/MM level. All

QM/MM simulations were performed with the QM/MM interface between Gromacs-4.5.3[12] and Gaussian03.[13]

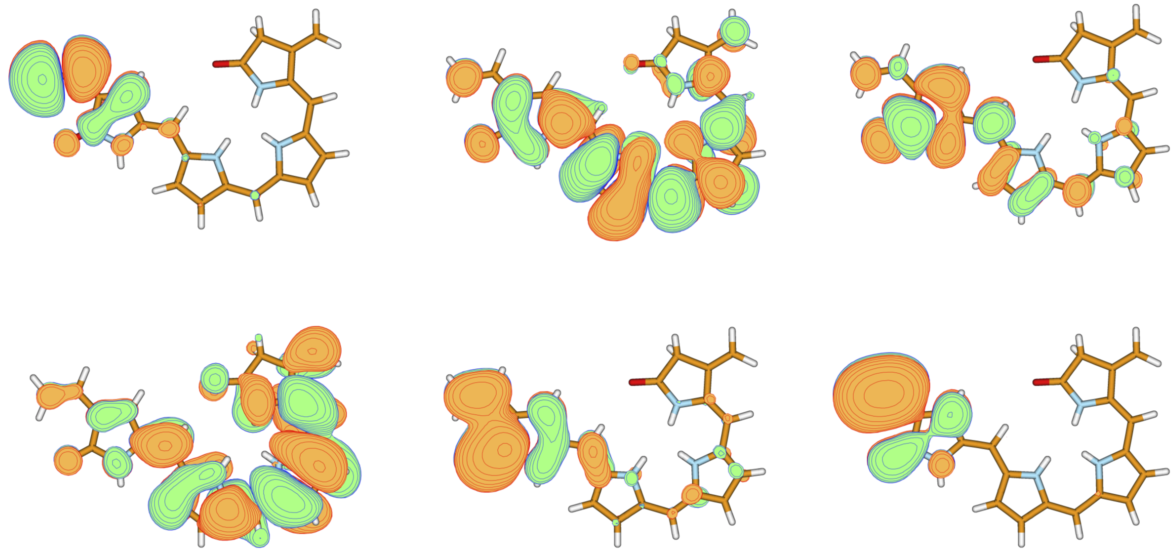

Figure S2: SA2-CASSCF(6,6)/3-21G active space orbitals used in the QM/MM MD simulations.

Analysis of the excited state trajectories

Table S1 summarizes the key results of all trajectories, including: (i) the excited state lifetime, defined as the time it takes to reach the conical intersection and deactivate; (ii) the final configuration of the chromophore at 1 ps after deactivation; and (iii) the time point at which the chromophore adopts the more planar early Lumi-R configuration.

Validation of chromophore QM model

To validate the SA2-CASSCF(6,6)/3-21G level of theory for the chromophore, we have recalculated the potential energies of the four trajectories that reach the ZZE photoproduct state (Trajectories 6, 11, 35 and 38, Table S1) at the highly-correlated xMCQDPT2/SA3-CASSCF(12,12)/cc-pVDZ level of theory.[14] Snapshots until 30-50 steps after deactivation at the conical intersection

Table S1: Overview of the excited state lifetimes (2nd column), photo-products (3rd column) and time points at which early Lumi-R is formed. (4th column) in 50 QM/MM trajectories.

Trajectory	S_1 lifetime	Geom.	Lumi-R
1	>5 ps	-	
2	767 fs	ZZZ	
3	>5 ps	-	
4	313 fs	ZZZ	
5	122 fs	ZZZ	
6	90 fs	ZZE	~ 26.5 ps
7	141 fs	ZZZ	
8	215 fs	ZZZ	
9	>5 ps	-	
10	240 fs	ZZZ	
11	147 fs	ZZE	~ 11 ps
12	411 fs	ZZZ	
13	>5 ps	-	
14	272 fs	ZZZ	
15	>5 ps	-	
16	309 fs	ZZZ	
17	246 fs	ZZZ	
18	153 fs	ZZZ	
19	>5 ps	-	
20	>5 ps	-	
21	400 fs	ZZZ	
22	>5 ps	-	
23	146 fs	ZZZ	
24	2009 fs	ZZZ	
25	260 fs	ZZZ	

Trajectory	S_1 lifetime	Geom.	Lumi-R
26	122 fs	ZZZ	
27	330 fs	ZZZ	
28	250 fs	ZZZ	
29	610 fs	ZZZ	
30	>5 ps	-	
31	>5 ps	-	
32	221 fs	ZZZ	
33	>5 ps	-	
34	>5 ps	-	
35	62 fs	ZZE	~ 14 ps
36	205 fs	ZZZ	
37	152 fs	ZZZ	
38	183 fs	ZZE	~ 31 ps
39	>5 ps	-	
40	408 fs	ZZZ	
41	110 fs	ZZZ	
42	>5 ps	-	
43	>5 ps	-	
44	305 fs	ZZZ	
45	78 fs	ZZZ	
46	>5 ps	-	
47	>5 ps	-	
48	414 fs	ZZZ	
49	157 fs	ZZZ	
50	202 fs	ZZZ	

were extracted from these trajectories. For each snapshot we recomputed the QM/MM energies of the S_0, S_1 and S_2 states.

The recomputed energy profiles along the photo-isomerization pathway are shown in Figure S3. In addition, we compare the energy gaps between the S_1 and S_0 at the SA2-CASSCF(6,6)/3-21G and xMCQDPT2/SA3-CASSCF(12,12)/cc-pVDZ levels of theory in Figure S4. For all trajectories the validation confirms that surface hops occurred around the points where energy gap between S_1 and S_0 states is sufficiently small (lower than 25 kJ/mol or 0.01 a.u.). The validation furthermore confirms that the S_2 state is neither directly involved in the excited-sate dynamics, nor crosses with S_1 state.

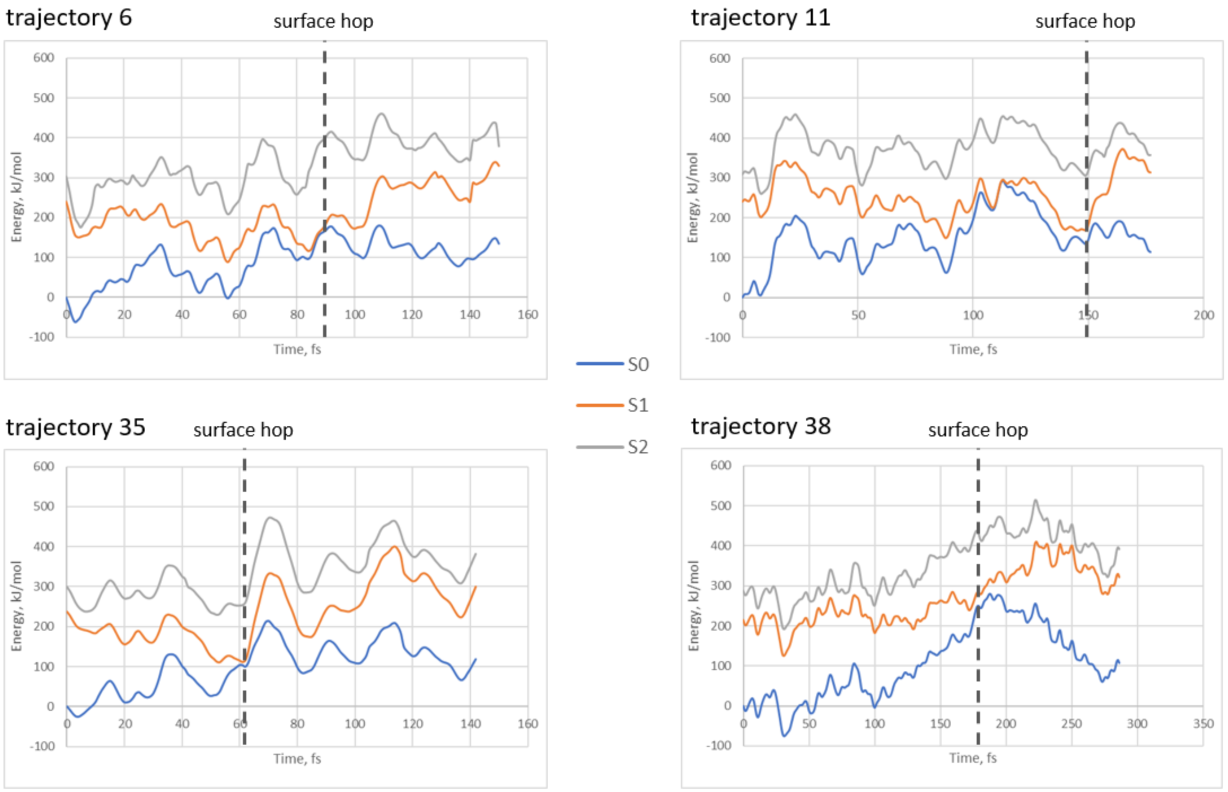

Figure S3: Recomputed energies of the successful isomerization trajectories at the xMCQDPT2/SA3-CASSCF(12,12)/cc-pVDZ//Amber03 level of theory. Dashed black lines correspond to the surface hop in the SA2-CASSCF(6,6)/3-21G//Amber03 simulations. All energies are plotted with respect to the potential energy of ground (S0) state at the start of the simulation.

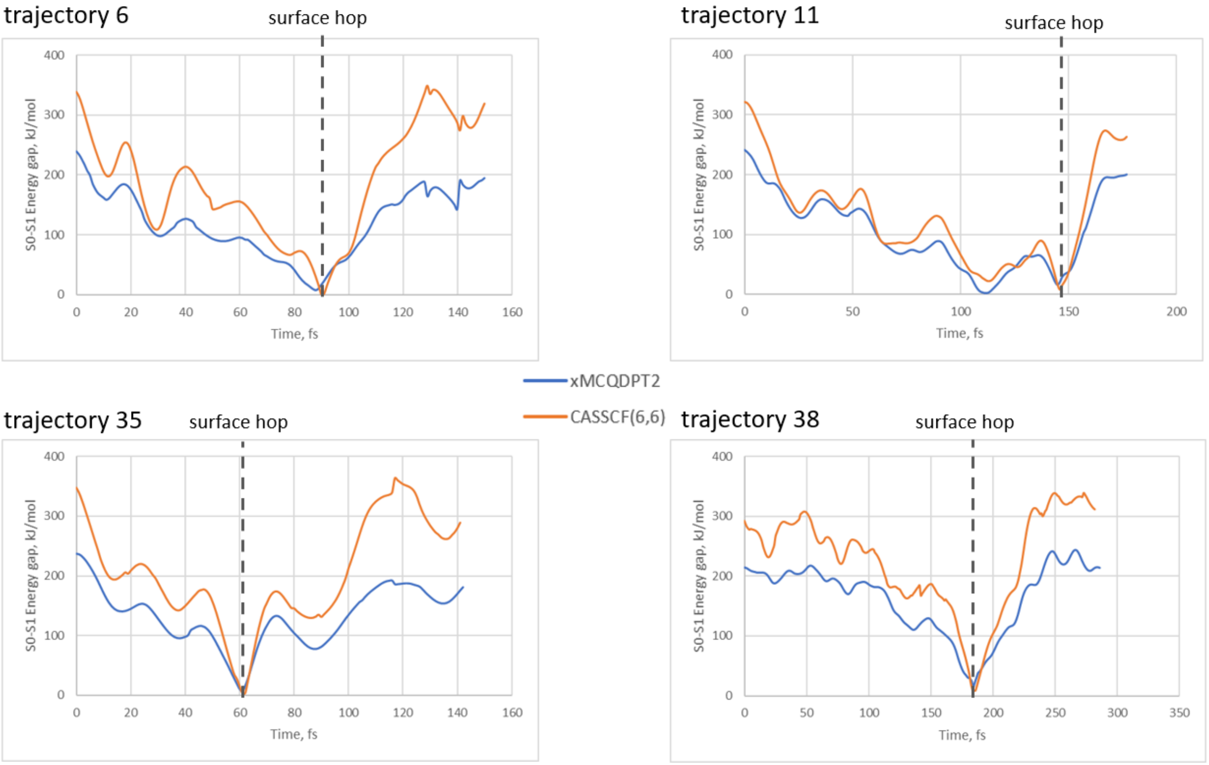

Figure S4: Energy gaps between the S_1 and S_0 states at the SA2-CASSCF(6,6)/3-21G//Amber03 (orange line) and xMCQDPT2/SA3-CASSCF(12,12)/cc-pVDZ//Amber03 (blue line) levels of theory in the successful isomerization trajectories. Dashed black lines correspond to the surface hop in the SA2-CASSCF(6,6)/3-21G//Amber03 simulations.

QM/MM geometry optimization

The QM/MM potential energy profile associated with the transition from the α_f to β_f disposition of the D ring was obtained by performing a 10-step relaxed potential energy scan along the angle between the planes formed by atoms C3C-C4C-C1D and C4C-C1D-C2D (see Figure S6 for atoms naming). The starting structure was taken from the relaxed ground state QM/MM MD trajectory after the excited state decay with a planar α_f disposition of D ring (early Lumi-R state). At each step of the QM/MM scan, the chromophore moiety was optimized at the DFT level, using the PBE[15] in combination with the DZVP basis set.[16] During these optimizations, the angle between the planes was restrained. The rest of the system, including the protein and water solvent, were

optimized with the Amber03 forcefield. The hybrid QM/MM geometry optimization calculations were carried out with CP2K.[17–19]

QM/MM umbrella sampling simulations

To include entropic effects on the estimate of the barrier for the α_f to β_f transition, we also performed umbrella sampling simulations,[20] according to the following protocol:

- First, we identified the angle defined by CMD-C1D-C4C-CMC (Figure S6) as the reaction coordinate for the α_f to β_f transition. The choice for this reaction coordinate was motivated by the structural differences of the chromophore between the early Lumi-R state and the $\mathrm{P_{fr}}$ state, which are largely captured by this reaction coordinate. We note, however, that because this is an approximation to the true reaction coordinate, the computed barrier may be an upper bound estimate of the true barrier. We performed an MD scan along the reaction coordinate using a time-dependent harmonic retraining potential. The center of this harmonic potential was slowly shifted toward product state at each MD step. The rate of this shift was constant during the simulation. We selected its value such that the transition is completed within 15-20 ps of MD simulation. This simulation is nearly equivalent to steered MD with a constant force. The canonical sampling through velocity rescaling (CSVR) thermostat[21] with strong coupling to the system was used at this stage to avoid overheating of the QM subsystem. The time constant of the CSVR thermostat during the scan was 1 fs.

- In the second step, frames from the MD scan trajectory were selected as starting points for 13 umbrella sampling simulations. A harmonic umbrella potential ($V = \frac{1}{2}k(\theta - \theta_0)^2$) with $k = 140$ kcal mol^{-1} was applied to all windows, with θ_0 ranging from -60 to 60 degrees with 10 degree increments.

- Third, a short biased MD simulation of 3-5 ps was performed to equilibrate each window. During this equilibration we used the CSVR thermostat with a 1 fs time constant.

- In the final stage, 30 ps trajectories were computed for each umbrella, starting from the coordinates and velocities at the last step of the equilibration runs. To keep the temperature constant at 300 K, the Nosé-Hoover thermostat chain[22] was used with a time constant of 1000 fs. The values of the reaction coordinate, θ, were saved at every MD step.

The Umbrella Sampling simulations were performed with CP2K,[17] in the NVT ensemble, at $T = 300$ K, and with a 1 fs timestep. The QM subsystem used in these simulations is shown in Figure S5 and includes chrompohore's conjugated π-system, the pyrole water molecule that is hydrogen-bound to NH hydrogens of A-C rings, and the side chain of Tyr277. We used the PBE functional,[15] and a short-range version of the DZVP basis set, which was optimized for molecular simulations.[16] The MM subsystem was modeled with the Amber03 force-field.[1]

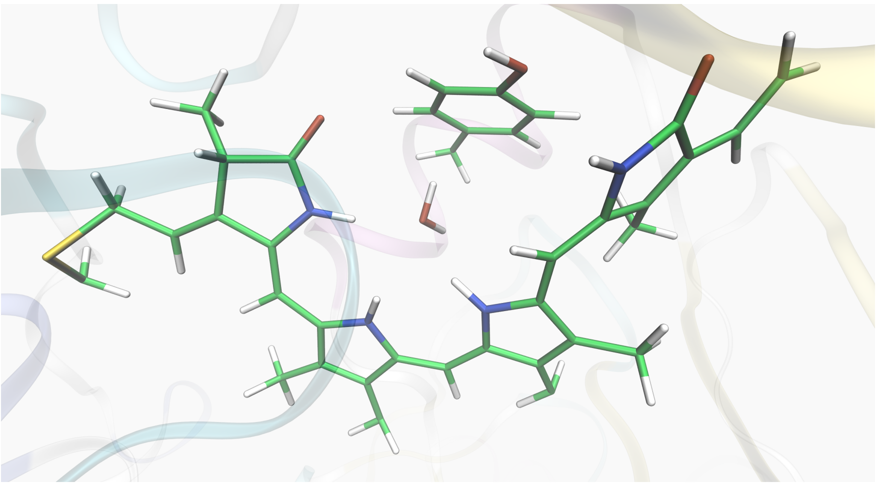

Figure S5: The QM subsystem in the umbrella sampling simulations.

We performed three sets of umbrella sampling simulations, each set starting in a different region of configuration space. The first set of US simulations was initiated from the last frame of the non-adiabatic MD trajectory, corresponding to an non-relaxed early Lumi-R state. For the second set of simulations, this early Lumi-R starting structure was equilibrated for 10 ns at the MM level at 1 bar and 300 K with the Gromacs package.[12] The third set of simulations was performed in the opposite direction by starting from a putative late Lumi-R conformation that was equilibrated for 10 ns at the MM level at 1 bar and 300 K with the Gromacs package.[12]

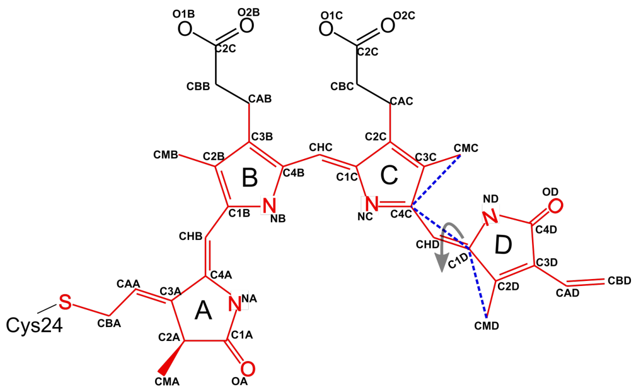

Figure S6: The dihedral angle used as reaction coordinate in umbrella sampling simulations of the α_f to β_f transition in the chromophore is indicated by the gray arrow.

The umbrella sampling simulations were analyzed with the weighted histogram analysis method (WHAM)[23] and umbrella integration (UI) method[24]. We used the WHAM program developed by Grossfield group,[25] and our own Python script for UI analysis, which is available from the GitHub.[26]

The probability densities of the reaction coordinate in each of the US windows are shown in Figure S7 and the unbiased potential of mean force (PMF) profiles are shown in Figure S8. While the UI and WHAM free energy energy profiles are nearly identical, the UI profiles are less noisy.

The differences between the PMF profiles in Figure S8 reveal that relaxation of the protein environment has an important effect on the calculated free energy profile of the α_f to β_f transition. Without relaxation, the barrier is 71 kJ/mol (Figure S8a) and the β_f disposition has a higher free energy that the α_f disposition of the D ring. In contrast, after relaxation, the barriers are significantly lower, but depend on whether we equilibrated the system in early or late Lumi-R state. We therefore combined the umbrellas of the latter two sets of simulations into one new set. In particular, we used the umbrella windows from -60 to -10 degrees of the set of simulations starting in the relaxed early Lumi-R state and the umbrella windows from 10 to 60 degrees of the set of simulations starting in the relaxed late Lumi-R state. The central umbrella was obtained by combining the umbrella windows at 0 degrees of both sets. The combined PMF profile, shown in Figure S8 has a barrier of 33 kJmol^{-1}.

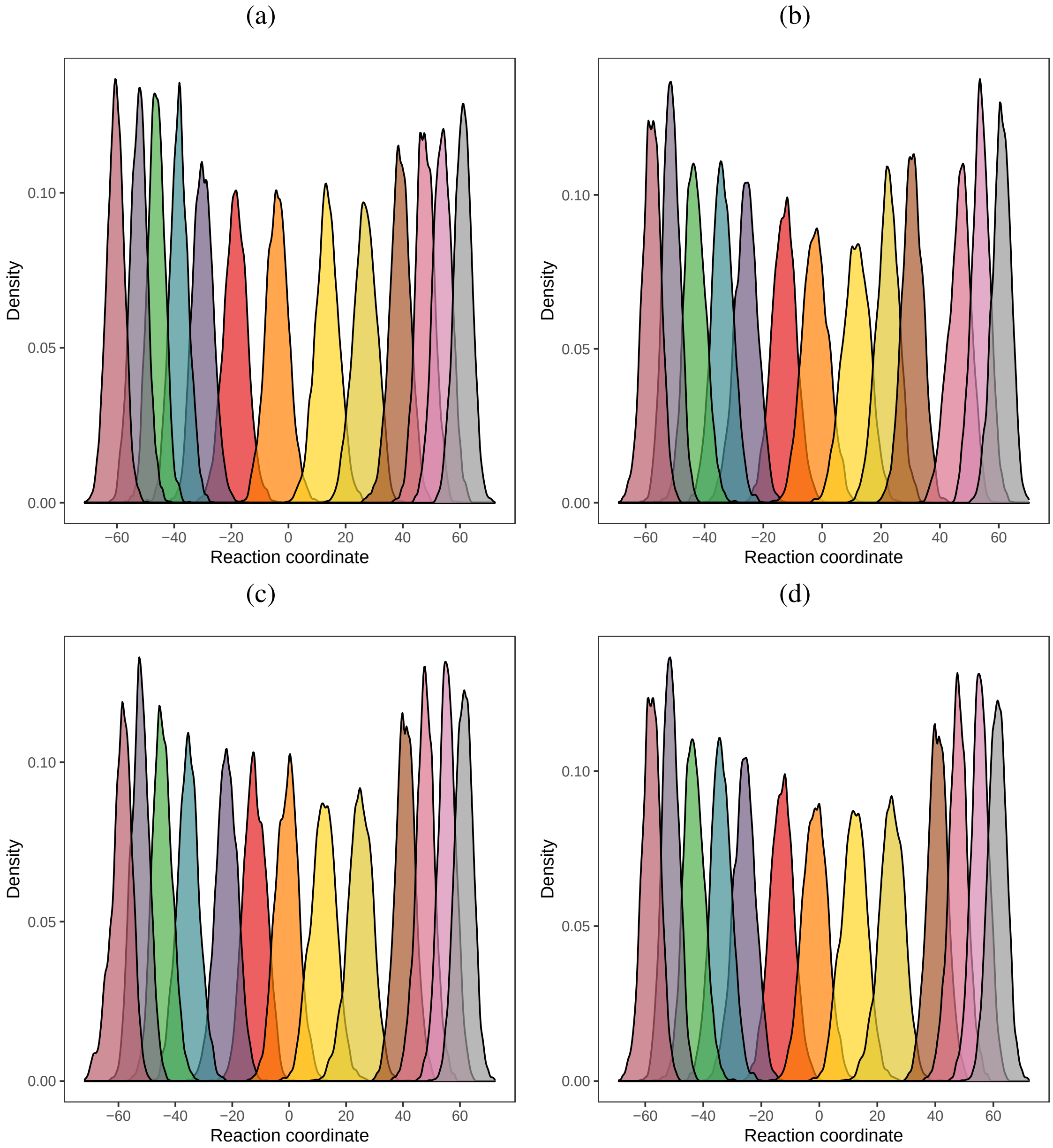

Figure S7: Density plots in the US windows in three sets of simulations: (a) simulations starting from the final frame of the non-adiabatic QM/MM trajectory; (b) simulations starting from a relaxed early Lumi-R state; (c) simulations starting from a relaxed late Lumi-R state. (d) combined windows from (b) and (c), see text for details.

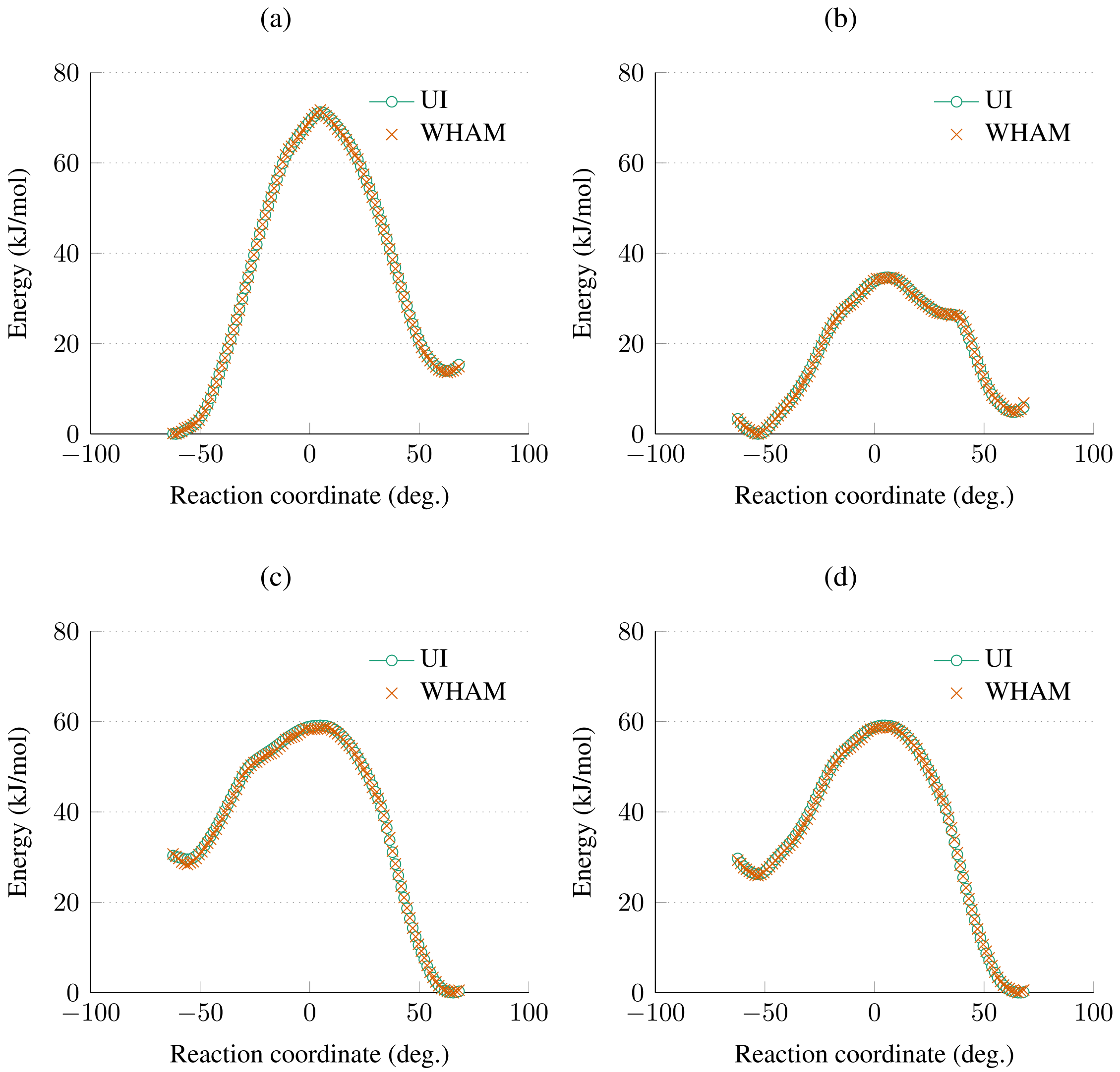

Figure S8: PMF profiles for the α_f to β_f transition of of the chromophore in CBD-PHY obtained from: (a) simulations starting from the final frame of the non-adiabatic QM/MM trajectory; (b) simulations starting from a relaxed early Lumi-R state; (c) simulations starting from a relaxed late Lumi-R state; (d) combined windows from (b) and (c), see text for details.

Circular dichroism spectra of P_r, early and late Lumi-R

We extracted the structures of the P_r, early and late Lumi-R states of the biliverdin chromophore and pyrrole water from the molecular dynamics trajectories and optimized these structures using density functional theory with the PBE0 functional,[27] and cc-pVDZ basis set.[28] After optimization, we computed the ten lowest-energy electronic excited states using time-dependent density functional theory (TD-DFT) with the PBE0 functional and the cc-pVDZ basis set. These calculations were performed with the Gaussian03 program.[13] Using the excitation energies, transition dipoles and rotary strength from these calculations,[29,30] we built the CD spectra with GaussSum 3.0.[31] The optimized geometries, and their CD spectra are shown of Figure S9.

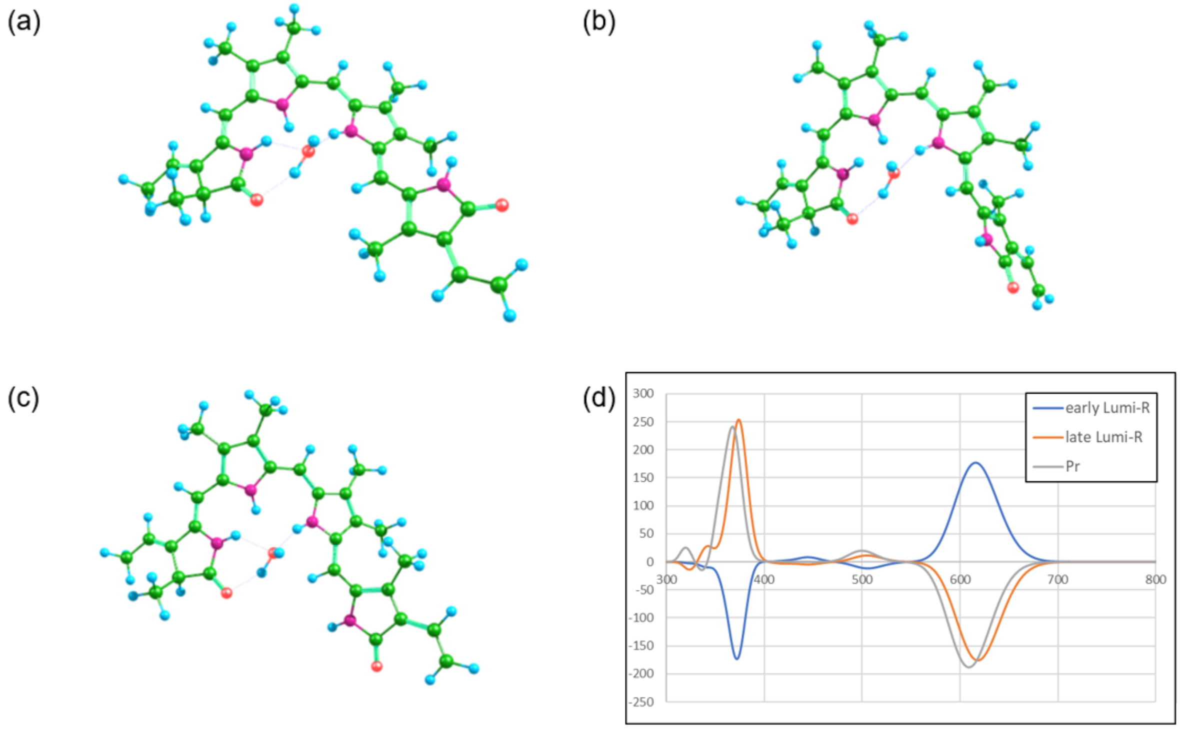

Figure S9: PBE0/cc-pVDZ optimized structures of the P_r (a), early Lumi-R (b) and late Lumi-R (c) conformations of the chromophore and their CD spectra (d).

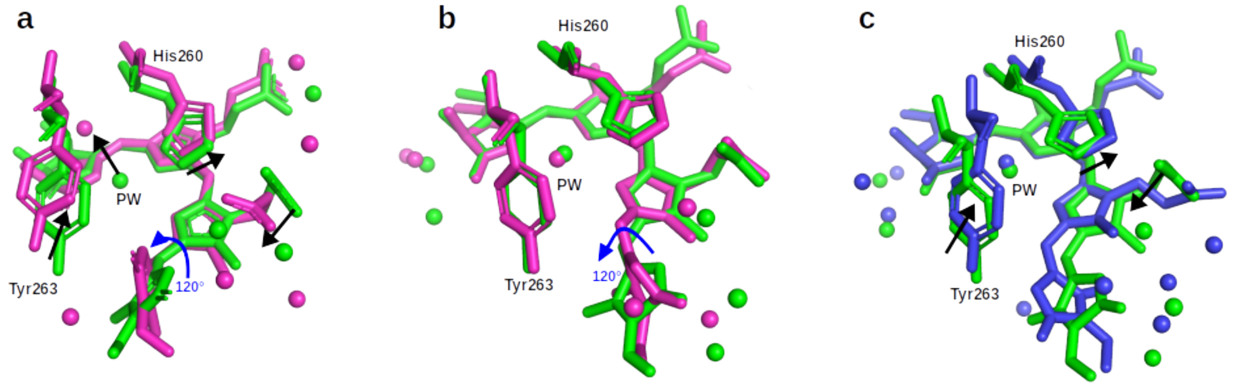

Figure S10: Structures of the chromophore pocket in the putative I_0 state (magenta) and early lumi-R state (blue) overlaid on the Pr resting state structure (green). Panel a: The refined 1 ps (magenta) and Pr (green) structures obtained from trSFX.[32] Panel b: I_0 intermediate (green) formed immediately after decay at the S_1/S_0 conical intersection in our QM/MM simulations. Panel c: the early Lumi-R intermediate (blue) at 15 ps later in our simulations. The most prominent structural changes are indicated by black arrows. The blue arrows indicate the rotation of the D ring by 120°. PW indicates the pyrrole water, which undergoes a much larger displacement in the refined structure compared to the MD snapshots.

References

(1) Duan, Y.; Wu, C.; Chowdhury, S.; Lee, M. C.; Xiong, G.; Zhang, W.; Yang, R.; Cieplak, P.; Luo, R.; Lee, T., et al. A point-charge force field for molecular mechanics simulations of proteins based on condensed-phase quantum mechanical calculations. *J. Comput. Chem.* **2003**, *24*, 1999–2012.

(2) Modi, V.; Donnini, S.; Groenhof, G.; Morozov, D. Protonation of the Biliverdin IXα Chromophore in the Red and Far-Red Photoactive States of a Bacteriophytochrome. *J. Phys. Chem. B.* **2019**, *123*, 2325–2334.

(3) Jorgensen, W. L.; Chandrasekhar, J.; Madura, J. D.; Impey, R. W.; Klein, M. L. Comparison of simple potential functions for simulating liquid water. *The Journal of chemical physics* **1983**, *79*, 926–935.

(4) Essmann, U.; Perera, L.; Berkowitz, M. L.; Darden, T.; Lee, H.; Pedersen, L. G. A smooth particle mesh Ewald method. *The Journal of chemical physics* **1995**, *103*, 8577–8593.

(5) Hess, B.; Bekker, H.; Berendsen, H. J.; Fraaije, J. G. LINCS: a linear constraint solver for molecular simulations. *Journal of computational chemistry* **1997**, *18*, 1463–1472.

(6) Miyamoto, S.; Kollman, P. A. SETTLE: An Analytical Version of the SHAKE and RATTLE Algorithms for Rigid Water Models. *J. Comp. Chem.* **1992**, *13*, 952–962.

(7) Warshel, A.; Levitt, M. Theoretical studies of enzymic reactions: dielectric, electrostatic and steric stabilization of the carbonium ion in the reaction of lysozyme. *J. Mol. Biol.* **1976**, *103*, 227–249.

(8) Boggio-Pasqua, M.; Burmeister, C. F.; Robb, M. A.; Groenhof, G. Photochemical reactions in biological systems: probing the effect of the environment by means of hybrid quantum chemistry/molecular mechanics simulations. *Phys. Chem. Chem. Phys.* **2012**, *14*, 7912–7928.

(9) Siegbahn, P. E.; Almlöf, J.; Heiberg, A.; Roos, B. O. The complete active space SCF (CASSCF) method in a Newton–Raphson formulation with application to the HNO molecule. *The Journal of Chemical Physics* **1981**, *74*, 2384–2396.

(10) Fabiano, E.; Keal, T.; Thiel, W. Implementation of surface hopping molecular dynamics using semiempirical methods. *Chemical Physics* **2008**, *349*, 334–347.

(11) Maximowitsch, E. Molecular mechanisms of spectral tuning and excited-state decay in phytochrome photoreceptors. Ph.D. thesis, 2020.

(12) Hess, B.; Kutzner, C.; Van Der Spoel, D.; Lindahl, E. GROMACS 4: algorithms for highly efficient, load-balanced, and scalable molecular simulation. *Journal of chemical theory and computation* **2008**, *4*, 435–447.

(13) Frisch, M. J.; Trucks, G. W.; Schlegel, H. B.; Scuseria, G. E.; Robb, M. A.; Cheeseman, J. R.; Montgomery, J. A., Jr.; Vreven, T.; Kudin, K. N.; Burant, J. C.; Millam, J. M.; Iyengar, S. S.; Tomasi, J.; Barone, V.; Mennucci, B.; Cossi, M.; Scalmani, G.; Rega, N.; Petersson, G. A.; Nakatsuji, H.; Hada, M.; Ehara, M.; Toyota, K.; Fukuda, R.; Hasegawa, J.; Ishida, M.; Nakajima, T.; Honda, Y.; Kitao, O.; Nakai, H.; Klene, M.; Li, X.; Knox, J. E.; Hratchian, H. P.; Cross, J. B.; Bakken, V.; Adamo, C.; Jaramillo, J.; Gomperts, R.; Stratmann, R. E.; Yazyev, O.; Austin, A. J.; Cammi, R.; Pomelli, C.; Ochterski, J. W.; Ayala, P. Y.; Morokuma, K.; Voth, G. A.; Salvador, P.; Dannenberg, J. J.; Zakrzewski, V. G.; Dapprich, S.; Daniels, A. D.; Strain, M. C.; Farkas, O.; Malick, D. K.; Rabuck, A. D.; Raghavachari, K.; Foresman, J. B.; Ortiz, J. V.; Cui, Q.; Baboul, A. G.; Clifford, S.; Cioslowski, J.; Stefanov, B. B.; Liu, G.; Liashenko, A.; Piskorz, P.; Komaromi, I.; Martin, R. L.; Fox, D. J.; Keith, T.; Al-Laham, M. A.; Peng, C. Y.; Nanayakkara, A.; Challacombe, M.; Gill, P. M. W.; Johnson, B.; Chen, W.; Wong, M. W.; Gonzalez, C.; Pople, J. A. Gaussian 03, Revision B.04. Gaussian, Inc., Wallingford, CT, 2004.

(14) Granovsky, A. A. Extended multi-configuration quasi-degenerate perturbation theory: The

new approach to multi-state multi-reference perturbation theory. *J. Chem. Phys.* **2011**, *134*, 214113.

(15) Perdew, J. P.; Burke, K.; Ernzerhof, M. Generalized gradient approximation made simple. *Physical review letters* **1996**, *77*, 3865.

(16) VandeVondele, J.; Hutter, J. Gaussian basis sets for accurate calculations on molecular systems in gas and condensed phases. *The Journal of chemical physics* **2007**, *127*, 114105.

(17) CP2K developers group, CP2K: A general program to perform molecular dynamics simulations, version 6.1. 2019; `www.cp2k.org`.

(18) Laino, T.; Mohamed, F.; Laio, A.; Parrinello, M. An efficient real space multigrid QM/MM electrostatic coupling. *Journal of Chemical Theory and Computation* **2005**, *1*, 1176–1184.

(19) Laino, T.; Mohamed, F.; Laio, A.; Parrinello, M. An efficient linear-scaling electrostatic coupling for treating periodic boundary conditions in QM/MM simulations. *Journal of chemical theory and computation* **2006**, *2*, 1370–1378.

(20) Torrie, G. M.; Valle, J. P. Non-physical sampling distributions in Monte-Carlo free energy estimation - umbrella sampling. *J. Comput. Phys.* **1977**, *23*, 187–199.

(21) Bussi, G.; Donadio, D.; Parrinello, M. Canonical sampling through velocity rescaling. *The Journal of chemical physics* **2007**, *126*, 014101.

(22) Nosé, S. A unified formulation of the constant temperature molecular dynamics methods. *The Journal of Chemical Physics* **1984**, *81*, 511.

(23) Kumar, S.; Rosenberg, J. M.; Bouzida, D.; Swendsen, R. H.; Kollman, P. A. The weighted histogram analysis method for free-energy calculations on biomolecules. I. The method. *Journal of computational chemistry* **1992**, *13*, 1011–1021.

(24) Kästner, J.; Thiel, W. Bridging the gap between thermodynamic integration and umbrella sampling provides a novel analysis method: "umbrella integration". *J. Chem. Phys.* **2005**, *123*, 1441041–1441045.

(25) Grossfield, A. WHAM: the weighted histogram analysis method, version 2.0.9.1. `http://membrane.urmc.rochester.edu/wordpress/?page_id=126`.

(26) V. Mironov, PyUI: A simple script to perform umbrella integration analysis. 2022; `https://github.com/vamironov/pyui`.

(27) Adamo, C.; Barone, V. Toward reliable density functional methods without adjustable parameters: The PBE0 model. *The Journal of chemical physics* **1999**, *110*, 6158–6170.

(28) Dunning Jr, T. H. Gaussian basis sets for use in correlated molecular calculations. I. The atoms boron through neon and hydrogen. *The Journal of chemical physics* **1989**, *90*, 1007–1023.

(29) Bauernschmitt, R.; Ahlrichs, R. Treatment of electronic excitations within the adiabatic approximation of time dependent density functional theory. *Chem. Phys. Lett.* **1996**, *256*, 454–464.

(30) Autschbach, J.; Ziegler, T.; van Gisbergen, S. J.; Baerends, E. J. Chiroptical properties from time-dependent density functional theory. I. Circular dichroism spectra of organic molecules. *The Journal of chemical physics* **2002**, *116*, 6930–6940.

(31) O'boyle, N. M.; Tenderholt, A. L.; Langner, K. M. Cclib: a library for package-independent computational chemistry algorithms. *Journal of computational chemistry* **2008**, *29*, 839–845.

(32) Claesson, E.; Wahlgren, W. Y.; Takala, H.; Pandey, S.; Castillon, L.; Kuznetsova, V.; Henry, L.; Panman, M.; Carrillo, M.; Kübel, J., et al. The primary structural photoresponse of phytochrome proteins captured by a femtosecond X-ray laser. *Elife* **2020**, *9*, e53514.
