# Supplementary material for: The Photocycle of Bacteriophytochrome Is Initiated by Counterclockwise Chromophore Isomerization
Source: J Phys Chem Lett. 2022 May 16;13(20):4538–42. doi: 10.1021/acs.jpclett.2c00899 (PMC9150100; doi:10.1021/acs.jpclett.2c00899)
Supplement: Supplementary file 5 — jz2c00899_si_005.pdf [file jz2c00899_si_005.pdf]

jz-2022-008994.R1

Name: Peer Review Information for "The Photocycle of Bacteriophytochrome Is Initiated by Counter-Clockwise Chromophore Isomerization"

First Round of Reviewer Comments

Reviewer: 1

Comments to the Author

The manuscript by *Morozov et al* focuses on the photoactivation of the *Deinococcus radiodurans* bacteriophytochrome, using QM/MM molecular dynamics simulation on the photosensory protein dimer to reach the first reaction intermediate, namely the Lumi-R, and then QM/MM Umbrella Sampling to reach the other Meta-R intermediate.

As the title suggests, the major outcome of this work is the counterclockwise rotation of the chromophore D-ring upon excitation to the S_1 electronic excited state. The other main finding concerns the Lumi-R-to- Meta-R conversion, for which the authors provide an energy barrier of 33 kJ/mol.

Although the messages of this work may be of interest for the scientific community, I believe that the paper does not present the urgency and novelty which are required for publication in the JPCL. The main point here is that a large part of the main results not only have been already presented in previous studies (ref. 16, and the recent ref. 29), but they are also not strongly supported by the applied methodology. In fact, the authors often need to use what found in the recent computational study reported in ref. 29 to give robustness to their findings.

Before proceeding further, the authors should also consider the following points:

1. Concerning the photochemical process, 50 trajectories, of which only 4 reach the photoproduct, are not enough to fully characterize a process that occurs with a quantum yield of the order of 10%, even considering the level of theory used for the chromophore (CASSCF(6,6)/3-21G). Moreover, it would be interesting to further investigate why 17 trajectories have excited-state lifetimes larger than 5 ps to understand if these are due to some clash or electrostatic interactions with the environment.

2. After the photochemical process, the authors carry out a ground-state molecular dynamics simulation of the picosecond time scale and, finally, QM/MM Umbrella Sampling. However, there is experimental evidence that there are two Lumi-R intermediates, which differ in terms of intermolecular features. Both ref. 39 and a recent paper (Kübel, J. et al. Phys. Chem. Chem. Phys. 2020, 22, 9195-9203) suggest that the Lumi-R intermediate evolves on a nanosecond time scale. Thus, investigating the Lumi-R to Meta-R process with a different binding pocket could decrease the energy barrier and/or provide a different reaction path. Therefore, I advise the authors to carry out a longer ground-state molecular dynamics before moving to the search of the Meta-R intermediate.

3. Concerning the Meta-R intermediate, the QM/MM umbrella sampling approach is surely interesting as it allows one to estimate an energy barrier of 33 kJ/mol. However, this result is strongly biased by the choice of the CV (the dihedral angle CMD-C1D-C4C-CMC). Since there isn't any experimental or computational evidence of how the Meta-R is reached, the authors should investigate in more details if what they provided is the only possible reaction path followed by the system. This analysis is also connected to the issue reported in the previous point.

Finally, I suggest the authors to focus more on these aspects:

1. Unlike ref. 29, where the authors studied only one monomer, this manuscript investigates the entire dimer. This is a strong point, on which the authors should insist more. In fact, they confirm that the direction of rotation is independent of the type of construct (dimer or monomer).

2. They report a hydrogen bonding interaction with the Tyr263 residue in the Lumi-R intermediate without stressing its importance. On the contrary, it seems that this interaction is of considerable interest since it could explain the role of such residue, highlighted in the literature, in the photoactivation mechanism (Takala, H. et al. J. Biol. Chem. 2018, 293, 8161- 8172).

Reviewer: 2

Comments to the Author

The research article entitled "The Photocycle of Bacteriophytochrome is Initiated by Counter-Clockwise Chromophore Isomerization", presents a computational dynamics study of the photo-activation of a bacteriophytochrome, including its photo-isomerization of the biliverdin chromophore. This article uses hybrid Quantum Mechanics / Molecular Mechanics and and non-adiabatic molecular dynamics simulations on a realistic photo-sensory protein dimer to resolve the isomerization mechanism in atomic detail.

1. What is the major advance reported in the paper?

• The phytochrome chromophore moiety simulation using hybrid QM/MM non-adiabatic MD simulations.

• The structural conforrmational change of the cofactor biliverdin within the time scales of sub-pico second and picosecond was determined.

• During the D-ring isomerization, the rotation of the D-ring rotates counter-clockwise. Assignment (tentative) of chromophore adopting the Pfr state conformation (ZZEssa) is observed, while the protein is (still) in Pr conformation.

2. What is the immediate significance of this advance?

• This work aligns with measured D-ring rotation using time resolved X-ray diffraction method. Further, it may provide a platform analyzing and rationalizing future phytochrome time resolved experiments. Modelling of the isomerization is a difficult task and this paper is the most complete in archiving it.

* The work suggest a mechanism to the observed structural changes; connecting protein structure, dynamics and function. It is an important contribution to the literature on computational investigations of phytochromes.

• In the structure, chromophore has already adopted configuration of the Pfr state (i.e., ZZEssa configuration), while the protein is still in the Pr conformation; this is very interesting.

3. Technical suggestions

• The pyrole water displacement (Claesson elife 2020) could have been taken consideration to show at what time scale this event happens. It should be discussed how this observed change can be rationalized.

• The author should compare the angle of rotation between the presented result and the trSFX structure in the elife paper.

• The photo-isomerization of the D-ring is around 120 degree (rotation) by 3ps-4ps. Is this photo-isomerization of the D-ring restricted to 120 degree due to molecular restraint of the BV or the water network around? Why does it not proceed further? Is it reasonable that the ring relaxes furthers until it reaches the Pfr state at 180 deg?

• Further, authors could discuss more how His260 (pi..pi) interaction with the C-ring of the BV influences (or may influence) the isomerization reaction. Charge transfer states have been proposed as primary states - this could be discussed in greater detail.

Author's Response to Peer Review Comments:

Dear Prof. Editor,

We thank both reviewers and you for the time and effort invested in the evaluation of our manuscript “The Photocycle of Bacteriophytochrome Is Initiated by Counter-Clockwise Chromophore Isomerization”. We were pleased that the reviewers found our work of high significance. We were also pleased that Reviewer 2 considers our work of high urgency and novelty. Unfortunately, Reviewer 1, did not share that opinion, because (i) a previous experimental study had already hinted at the rotation of the D-ring in the counter-clockwise direction (Claeson et al, ref. 16), and (ii) a preprint of another computational study had appeared in which the same problem was addressed with a different simulation methodology (Salvadori et al, ref. 29). We speculate, however that the disagreement between the reviewers concerning the novelty and urgency, might be partially due to a misunderstanding by reviewer 1 of the relation between our manuscript and the previous experimental work (ref. 16) and computational work (ref. 29)

Because we were involved in the experimental study (ref. 16), we are aware of the findings presented in ref. 16 and their relation to the results presented in our current manuscript. Indeed, our main motivation for the computational work described in this manuscript, was to address the most important open questions that our experimental data could not resolve, including (i) whether the structure we reported is a ground or excited state intermediate in the isomerization process, (ii) what is the rotation mechanism, and (iii) how does the highly strained intermediate evolve further into the Lumi-R conformation? Because the results of our simulations provide answers to these important questions, we believe that our work has sufficient novelty and urgency to be shared with the broader readership of the *Journal of Physical Chemistry Letters*.

While we acknowledge that a preprint describing similar results had appeared on ChemRXiv (on the 9th of March) before ours was submitted, we’d like to emphasize that this happened while we were completing our submission. Because this preprint appeared, however, we have delayed our submission by a few more days in order to revise our manuscript and discuss our results in relation to those described in that preprint (ref. 29). Eventually, we submitted our manuscript on the 18th of March, initially to the *Journal of the American Chemical Soc*iety, from where it was transferred to the *Journal of Physical Chemistry Letters* ten days later). We therefore do not share the opinion of the reviewer that the preprint would diminish the novelty or urgency of our work.

Nevertheless, both reviewers also raised interesting questions as well as valuable suggestions to improve the presentation of our results. We have addressed their comments, answered their questions and revised our manuscript accordingly. On the following pages, you will find our detailed responses to the comments of both reviewers.

We hope that with the changes based on the reviewers' comments, our manuscript will be suitable for publication in the *Journal of Physical Chemical Letters*.

On behalf of all authors,

Dmitry Morozov and Gerrit Groenhof

Reviewer 1

Comment 1: The manuscript by Morozov et al focuses on the photoactivation of the Deinococcus radiodurans bacteriophytochrome, using QM/MM molecular dynamics simulation on the photosensory protein dimer to reach the first reaction intermediate, namely the Lumi-R, and then QM/MM Umbrella Sampling to reach the other Meta-R intermediate.

As the title suggests, the major outcome of this work is the counterclockwise rotation of the chromophore D-ring upon excitation to the S1 electronic excited state. The other main finding concerns the Lumi-R-to- Meta-R conversion, for which the authors provide an energy barrier of 33 kJ/mol.

Although the messages of this work may be of interest for the scientific community, I believe that the paper does not present the urgency and novelty which are required for publication in the JPCL. The main point here is that a large part of the main results not only have been already presented in previous studies (ref. 16, and the recent ref. 29), but they are also not strongly supported by the applied methodology. In fact, the authors often need to use what was found in the recent computational study reported in ref. 29 to give robustness to their findings.

Response 1: We thank the reviewer for the time and effort spent in evaluating our manuscript. The reviewer is of the opinion that our work is not sufficiently novel or urgent because the main findings, namely that the isomerization of the chromophore D ring proceeds in a counter-clockwise manner, had already been suggested in a previous experimental study (Cleason et al. Elife 9 (2020) e53514) and in a preprint (Salvadori et al.) that appeared on ChemRxiv a few days before our manuscript was submitted. While we respect the reviewers' opinion, we do not understand how the experimental paper or the preprint would affect the novelty or urgency of our results. On the contrary, the results described in our manuscript provide a rationale for the observations described in Cleasson *et al*. (ref. 16) that could not be explained based on the experimental data alone. Furthermore, as we explain below, our results complement those reported by Salvadori (ref. 29) reinforcing the main conclusions of both computational studies about the isomerization mechanism.

As co-authors of Claesson *et al*., (ref. 16) we are fully aware that our experimental findings raised several important questions that we could not

resolve based on our data: (i) What is the mechanism of rotation? (ii) Is the structure that we could refine, an intermediate on the electronic ground (S_0) or excited (S_1) state? (iii) How does this intermediate relax further into the first spectroscopically identifiable lumi-R state? Because the results of our multi-scale MD simulations suggest answers to each of these open questions, we consider our work both novel and urgent.

While Salvadori et al. (ref. 29) have also investigated the isomerization mechanism by means of multi-scale MD simulations to reach the same conclusion as us about the direction of the D-ring isomerization, they used the chromophore binding domain (CBD) monomer and a semi-empirical QM method, whereas we used the more complete CPB-PHY dimer and an ab initio method, which we furthermore validated at one of the highest levels of theory possible for such complex system. In our opinion, therefore, the two manuscripts complement each other and reinforce the important message that isomerization happens on a sub-ps timescale and in the counter-clockwise direction. Observing the same isomerization mechanism with different methods (CASSCF versus AM1), and in different protein environments (CBD monomer versus CBD-PHY dimer), strengthens the significance of these findings. Because the mechanism found in our simulations is in contrast to previous suggestions based on circular dichroism spectra of the dark and illuminated states, we also anticipate that both computational works will prompt new experiments. Finally, we would like to emphasize that the preprint by Salvadori *et al.* (ref. 29) appeared on ChemRXiv while we were completing our submission. In order to give proper credit to Salvadori et al., and to discuss our findings in the context of theirs, we had to revise our manuscript, which further delayed our submission. We therefore are of the opinion that the preprint by Salvadori et al., which to the best of our knowledge has not yet appeared as a peer-reviewed journal article, does not render our results less urgent or less novel.

The reviewer furthermore remarks that our results are not strongly supported by the applied methodology, but seems to base that opinion on the fact that we refer to the results in the preprint by Salvadori et al. to give robustness to our findings. Because we exclusively refer to the results by Salvadori et al. in the context of the photo-isomerization mechanism, we infer that the reviewer is referring to the methodology we employed to perform non-adiabatic QM/MM molecular dynamics simulations of the chromophore after photo-excitation. While the QM/MM simulations in ref. 29 relied on the semi-empirical AM1 hamiltonian, which to the best of our knowledge seems to have not been further

validated in that work, we used the *ab initio* CASSCF method. Because we used a truncated active space with 6 electrons in 6 orbitals, as well as the rather small 3-21G basis set, we have carefully validated the results of our MD simulations at the fully correlated xMCQDPT2/cc-pVDZ level of theory. These results, discussed in the Supporting Information of our manuscript, show that the time-evolution of the potential energy and energy gaps is highly similar at both levels of theory, which provides strong support for the validity of our CASSCF(6,6)/3-21G//Amber03 model for the phytochrome system. The high similarity between the results in ref. 29 and ours, in turn also provides a validation for the AM1 model employed in that work. Nevertheless, to avoid creating the impression that we need the results of ref. 29 to give robustness of our (independent!) findings, we have changed the way in which we refer to ref. 29 to emphasize more strongly the differences with that work.

Page 5: "..., which is in line with recent simulations of the CBD monomer[29]"

Comment 2: Before proceeding further, the authors should also consider the following points:

Concerning the photochemical process, 50 trajectories, of which only 4 reach the photoproduct, are not enough to fully characterize a process that occurs with a quantum yield of the order of 10%, even considering the level of theory used for the chromophore (CASSCF(6,6)/3-21G). Moreover, it would be interesting to further investigate why 17 trajectories have excited-state lifetimes larger than 5 ps to understand if these are due to some clash or electrostatic interactions with the environment.

Response 2: The reviewer remarks that 50 trajectories, of which 4 reach the photo-product state are insufficient to fully characterize a process that occurs with a quantum yield of 10%, but does not state how many trajectories would have been needed in order to do so. We therefore do not understand if the reviewer wants us to perform additional simulations or not. Nevertheless, we respectfully disagree that 50 trajectories would not be enough to support our conclusions that successful photoisomerization involves a counter-clockwise rotation of the D-ring and occurs on a sub-ps timescale, because even if the number of trajectories is statistically low, they yield consistent results, *i.e.,* the isomerization reaction follows the same pathway in all trajectories. Since the aim of our simulations was not to estimate quantum yields or excited state lifetimes, but to provide mechanistic insights into the isomerization process, we consider 50 trajectories that provide internally consistent mechanisms for the

three different processes (*i.e.*, (i) photo-isomerization towards Lumi-R, (ii) internal conversion back into the P_r state, and (iii) preservation of planarity without decay), sufficient to draw the qualitative conclusions on which our manuscript is based.

To alert the reader to the fact that the number of trajectories is low we have added following sentence:

Page 7: "Statistically, the number of trajectories is small, but nevertheless yields a consistent picture of the photo-isomerization mechanism."

The reviewer also writes "even considering the level of theory used for the chromophore (CASSCF(6,6)/3-21G)", but also here we may have not understood what the reviewer means. We speculate that the reviewer may be concerned about the accuracy of the level of theory? Because this is a concern that we also had when we started our simulations, we have carefully validated this level of theory by re-computing the potential energies of the photo-isomerization trajectories at the correlated xMCQDPT2/SA3-CASSCF(12,12)/cc-pVDZ//Amber03 level of theory. The good agreement between the potential energy profiles (Figure S3) and the energy gaps (Figure S4), at both levels of theory suggests that the CASSCF(6,6)/3-21G//Amber03 model provides a qualitatively correct description of the excited and ground state potential energy surfaces. We therefore consider that with the computational tools available today, we have sufficiently verified the validity of the level of theory we used to perform our non-adiabatic MD simulations.

To point out that we have validated our model at a higher level of correlated electronic structure theory, we added:

Page 3: "All details of the non-adiabatic simulations are provided as Supporting Information (SI), including a validation of our model at the correlated xMCQDPT2/SA3-CASSCF(12,12)/cc-pVDZ level of theory.[24]"

Finally, the reviewer would find it interesting if we can investigate why in 17 trajectories the chromophore remains planar and does not decay. We thank the reviewer for this suggestion and carefully inspected the interactions between the chromophore and the protein plus solvent. Unfortunately, there are no obvious visible differences between the starting structures that decay on the one hand, and the starting structures that do not decay on the other hand.

Therefore, even if we agree with the reviewer that this is an interesting suggestion, we consider this beyond the scope of our manuscript.

Comment 3: After the photochemical process, the authors carry out a ground-state molecular dynamics simulation of the picosecond time scale and, finally, QM/MM Umbrella Sampling. However, there is experimental evidence that there are two Lumi-R intermediates, which differ in terms of intermolecular features. Both ref. 39 and a recent paper (Kübel, J. et al. Phys. Chem. Chem. Phys. 2020, 22, 9195-9203) suggest that the Lumi-R intermediate evolves on a nanosecond time scale. Thus, investigating the Lumi-R to Meta-R process with a different binding pocket could decrease the energy barrier and/or provide a different reaction path. Therefore, I advise the authors to carry out a longer ground-state molecular dynamics before moving to the search of the Meta-R intermediate.

Response 3: Because there are (at least) two Lumi-R states separated by 100s of ns according to recent transient IR spectroscopy measurements, the reviewer is concerned that the lumi-R state from which we initiated our Umbrella Sampling simulations, may have not been equilibrated sufficiently, as we performed only short picosecond timescale ground-state QM/MM simulations after the photon-isomerization. The reviewer suggests therefore to perform longer MD simulations of the Lumi-R state, before searching for the Meta-R state. The reviewer further mentions a recent paper by Kübel *et al*. that reports on the formation of the aforementioned second Lumi-R intermediate on a ns-**μ**s timescale. Because we had overlooked this paper, we thank the reviewer for pointing us to this highly relevant paper. After reading it and discussing it with one of the authors (J. Ihalainen, personal communication), we concluded that what we had called Meta-R is more likely to be the second Lumi-R intermediate, identified by Kübel *et al*. Because transient spectroscopy in the UV/vis suggests the formation of a Lumi-R intermediate within 10s to 100s of picoseconds, we still attribute the first stable intermediate after I0 to this Lumi-R and call it "early Lumi-R". However, with a barrier of 33 kJ/mol for the transition from the α_f to β_f disposition of the D ring, this process would occur on a 62 ns time scale, in line with the rate of the formation of the second lumi-R state observed experimentally by Kübel *et al*. We therefore assign what we called "Meta-R" before to "late Lumi-R" in the revision. With a barrier of 33 kJ/mol, a classical MD simulation of several hundreds of nanoseconds, as suggested by the reviewer, would in principle suffice to observe the transition from early lumi-R into late lumi-R, but because the chromophore force-field was parameterized based on the configuration of the chromophore in the P_r and P_{fr} states, rather

than the lumi-R state, a re-parameterization of the force field would have been required. We deem such re-parameterization unnecessary, because the Umbrella Sampling at the more accurate QM/MM level already provides important mechanistic insights into the further relaxation of the chromophore towards the activated P_{fr} state.

To reflect the new insight, for which we again thank the reviewer, we have replaced Meta-R by late Lumi-R and added the following text:

Page 7: “Thus, based on Eyring's Transition State Theory (TST), the timescale of this inversion process would be in the order of 62 nanoseconds, much faster than the onset of the large protein structural changes seen in time-resolved WAXS experiments,[10] but qualitatively in line with the timescales at which a late Lumi-R state was observed in transient infra-red (trIR) spectroscopy measurements.[38,41]”

Comment 4: Concerning the Meta-R intermediate, the QM/MM umbrella sampling approach is surely interesting as it allows one to estimate an energy barrier of 33 kJ/mol. However, this result is strongly biased by the choice of the CV (the dihedral angle CMD-C1D-C4C-CMC). Since there isn’t any experimental or computational evidence of how the Meta-R is reached, the authors should investigate in more detail if what they provided is the only possible reaction path followed by the system. This analysis is also connected to the issue reported in the previous point.

Response 4: The reviewer is concerned that the free energy profiles depend too strongly on the reaction coordinate along which we have performed the QM/MM umbrella sampling simulations. While we agree that, in particular the height of the barrier can depend on the reaction coordinate, this barrier will always be overestimated, rather than underestimated if the reaction coordinate deviates from the ideal or “true” reaction coordinate. Therefore, the calculated barrier will always be an upper bound estimate to the true barrier.

Nevertheless, the choice for the CMD-C1D-C4C-CMC torsion as the reaction coordinate was based on the structural difference of the chromophore between the Lumi-R state found in our work, and the Pfr state, into which the system eventually would evolve. For this transition, which involves a change from the α_f to β_f disposition of the D ring, we consider the CMD-C1D-C4C-CMC torsion the most suitable reaction-coordinate, as this torsion constitutes the main structural difference between the two chromophore configurations. The validity

of our finding can in principle be verified by means of transient circular dichroism (CD) spectroscopy, as we suggest in the text.

To reflect that the choice of reaction coordinate can affect the barrier obtained from the Umbrella Sampling simulations and that the calculated barrier is therefore an upper bound estimate to the true barrier, we have added to a small discussion to the text:

Page 7: ”The results of these simulations, shown in Figure S8, suggest an upper bound of 33 $kJmol^{-1}$ for the barrier separating the α_f to β_f dispositions of the D ring.”

And

Page 9 in SI: “The choice for this reaction coordinate was motivated by the structural differences of the chromophore between the early Lumi-R state and the P_{fr} state, which are largely captured by this reaction coordinate. We note, however, that because this is an approximation to the true reaction coordinate, the computed barrier may be an upper bound estimate of the true barrier.”

Comment 5: Finally, I suggest the authors to focus more on these aspects: Unlike ref. 29, where the authors studied only one monomer, this manuscript investigates the entire dimer. This is a strong point, on which the authors should insist more. In fact, they confirm that the direction of rotation is independent of the type of construct (dimer or monomer).

Response 5: To help the reader distinguish between our work and that of ref. 29, the reviewer suggests to emphasize that we have performed simulations of the CBD-PHY dimer. We thank the reviewer for this good suggestion. We have now revised the text as follows:

Page 5: “The excited state decay process in these 33 trajectories takes less than a picosecond on average (Table S1), which is in line with recent simulations of the CBD monomer,[29]”

Comment 6: They report a hydrogen bonding interaction with the Tyr263 residue in the Lumi-R intermediate without stressing its importance. On the contrary, it seems that this interaction is of considerable interest since it could explain the role of such residue, highlighted in the literature, in the photoactivation mechanism (Takala, H. et al. J. Biol. Chem. 2018, 293, 8161-8172).

Response 6: The reviewer suggests to discuss in more detail the role of the highly conserved Tyr263 residue in stabilizing the Lumi-R intermediate, in particular, because the role of this residue has been highlighted in Takala *et al. J. Biol. Chem.* 293, (2018) 8161-8172 (now ref. 39). We thank the reviewer for drawing our attention to that paper, which we had overlooked. Because we indeed find that the hydrogen bond between Tyr263 and the D-ring stabilizes both the early and late Lumi-R states, we now emphasize the role of this residue more strongly by referring to the observation that replacing this residue by a phenylalanine hinders the formation of the Lumi-R state:

Page 6: “Because mutating this residue into a phenylalanine hinders the formation of Lumi-R,[39] we attribute the configuration in Figure 3b to the early Lumi-R state, which is also observed on similar timescales in transient absorption spectroscopy experiments.[28]“

Reviewer 2

Comment 1: The research article entitled “The Photocycle of Bacteriophytochrome is Initiated by Counter-Clockwise Chromophore Isomerization”, presents a computational dynamics study of the photo-activation of a bacteriophytochrome, including its photo-isomerization of the biliverdin chromophore. This article uses hybrid Quantum Mechanics / Molecular Mechanics and and non-adiabatic molecular dynamics simulations on a realistic photo-sensory protein dimer to resolve the isomerization mechanism in atomic detail.

1. What is the major advance reported in the paper?
   - The phytochrome chromophore moiety simulation using hybrid QM/MM non-adiabatic MD simulations.
   - The structural conformational change of the cofactor biliverdin within the time scales of sub-pico second and picosecond was determined.
   - During the D-ring isomerization, the rotation of the D-ring rotates counter-clockwise. Assignment (tentative) of chromophore adopting the Pfr state conformation (ZZEssa) is observed, while the protein is (still) in Pr conformation.

2. What is the immediate significance of this advance?
   - This work aligns with measured D-ring rotation using time resolved X-ray diffraction method. Further, it may provide a platform analyzing and rationalizing future phytochrome time resolved experiments. Modelling of the isomerization is a difficult task and this paper is the most complete in archiving it.
   - The work suggest a mechanism to the observed structural changes; connecting protein structure, dynamics and function. It is an important contribution to the literature on computational investigations of phytochromes.
   - In the structure, chromophore has already adopted configuration of the Pfr state (i.e., ZZEssa configuration), while the protein is still in the Pr conformation; this is very interesting.

3. Technical suggestions

Response 1: The reviewer provides an excellent overview of the main findings in our manuscript as well as their significance. We are pleased that the

reviewer appreciates our efforts and are thankful for the technical suggestions, which have helped us to improve the presentation of our results, as explained in detail below.

Comment 2: The pyrole water displacement (Claesson elife 2020) could have been taken into consideration to show at what time scale this event happens. It should be discussed how this observed change can be rationalized.

Response 2: The reviewer suggests to discuss the displacement of the pyrrole water molecule, which was observed to photo-dissociate from the chromophore in the trSFX experiments of Claesson *et al*. While the rotation of the D-ring and displacement of C-ring propionate, His260 and Tyr263 are in line with the structural changes reported by Claesson *et al*. (ref 16), we observe only a small displacement of the pyrrole water. We note that Salvadori *et al*. also did not observe a photo-dissociation in their QM/MM simulations.

While we do not know why the simulations suggest a much smaller displacement, we speculate that the much larger displacement observed by Claesson *et al*. might report on a competing pathway that was accessible during the tr-SFX experiment due to the very high photon flux of 5.48 10^{15} 640 nm photons per mm^2, which in principle could lead to multi-photon absorption (up to 58 per protein), ionizing the chromophore. Nevertheless, while our simulations do not provide a rationale for the photo-disociation of the pyrrole water molecule reported by Claesson et al., we do agree that this discrepancy should be discussed. We therefore have added a new figure to the Supporting information (Figure S10), in which we compare the structural differences in chromophore pocket reported by Cleasson *et al.,* to the structures of the I_0 and early Lumi-R structures obtained in our simulations. In addition, we added a line to the text to acknowledge that, in contrast to the refined SFX structures, we do not observe a large pyrrole water displacement.

Page 5: “A comparison between the twisted intermediate found in our simulations and the 1 ps structure refined by Claesson *et al.*[16] in Figure S10 (SI) reveals that the simulations predict a very similar chromophore configuration, but not the large displacement of the pyrrole water molecule. We speculate therefore that the photo-dissociation of the pyrrole water observed in trSFX, might have been induced by a multi-photon absorption process due to very high laser power in the experiments.”

And

Page 15 SI:

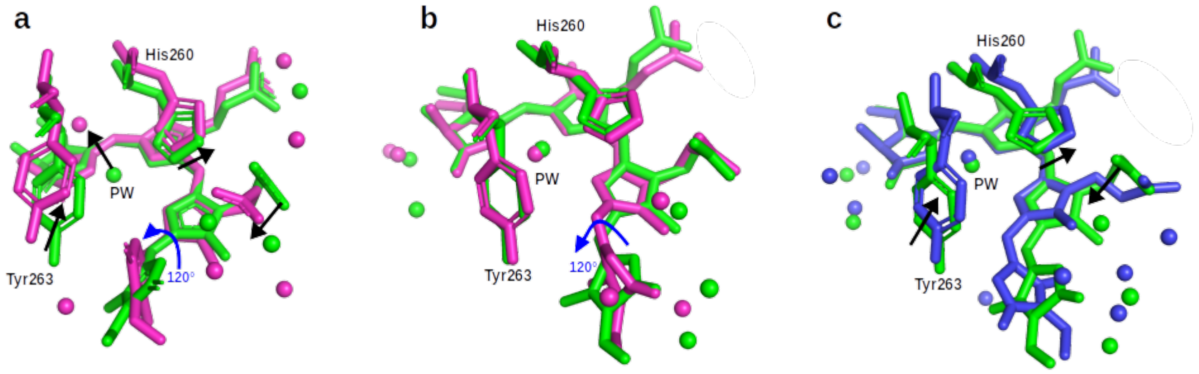

”Figure S10: Structures of the chromophore pocket in the putative I0 state (magenta) and early lumi-R state (blue) overlaid on the Pr resting state structure (green). Panel a: The refined 1 ps (magenta) and Pr (green) structures obtained from trSFX.[32] Panel b: I0 intermediate (green) formed immediately after decay at the S1/S0 conical intersection in our QM/MM simulations. Panel c: the early Lumi-R intermediate (blue) at 15 ps later in our simulations. The most prominent structural changes are indicated by black arrows. The blue arrows indicate the rotation of the D ring by 120 degrees. PR indicates the pyrrole water, which undergoes a much larger displacement in the refined structure compared to the MD snapshots.”

Comment 3: The author should compare the angle of rotation between the presented result and the trSFX structure in the elife paper.

Response 3: The reviewer suggests comparing the angle of rotation between our I_0 structure and the refined structure at 1 ps by Claesson *et al*. We agree that such comparison is useful and have added Figure S10 to the supporting information, in which we compare the chromophore pocket refined at 1 ps by Cleason *et al*. to the I_0 and Lumi-R structures obtained in our simulations. See our response to the previous comment for further details.

Comment 4: The photo-isomerization of the D-ring is around 120 degree (rotation) by 3ps-4ps. Is this photo-isomerization of the D-ring restricted to 120 degree due to molecular restraint of the BV or the water network around? Why does it not proceed further? Is it reasonable that the ring relaxes further until it reaches the Pfr state at 180 deg?

Response 4: The reviewer would like to understand why immediately after photo-isomerization the chromophore adopts a configuration in which the D-ring is rotated by 120 degrees. Visual inspection of the highly-twisted I_0 intermediate suggests that hydrogen bonds between the D-ring carbonyl and a cluster of water molecules stabilize this configuration (Figure 3a). Indeed, only after (spontaneous) disruption of this hydrogen bond network, does the D-ring continue isomerizing until the nitrogen group donates a hydrogen bond to the phenol group of the highly conserved Tyr263 (Figure 3b).

The reviewer also wants to know if it is reasonable that the D ring relaxes further until it reaches the Pfr state at 180 degrees. We consider this reasonable, but to reach the 180-degree rotation angle, the D ring has to change its disposition from the α_f to β_f disposition, which involves a barrier with an upper bound of 33 kJ/mol.

To emphasize these two important points, we have changed the caption of Figure 3:

Page 6: “We assign this configuration, in which the D-ring has undergone a 180 degree rotation with respect to the Pr resting state, to the late Lumi-R state.[38]”

Comment 5: Further, authors could discuss more how His260 (pi..pi) interaction with the C-ring of the BV influences (or may influence) the isomerization reaction. Charge transfer states have been proposed as primary states - this could be discussed in greater detail.

Response 5: The reviewer suggests that we discuss in more detail how His260, which is interacting with the C-ring of the chromophore via π-π stacking interactions, may influence the isomerization reaction. The reviewer furthermore suggests that charge transfer states had been proposed as primary states and also asks us to discuss this. Before commenting on the reviewer’s remark about the involvement of charge transfer states, we note that because the His260 side chain was not included inside the QM region, which contained only the chromophore, we could only model the electrostatic and steric interactions between His260 and the chromophore, but not charge transfer interactions. Because isomerization takes place at the D ring, rather than the C ring, we speculate, however, that the interaction between His260 and the C ring of the chromophore can only influence the isomerization process by restraining the orientation of the C ring.

Unfortunately, the reviewer did not include references to papers describing the involvement of charge transfer (CT) states. After some searching we did find the PhD thesis of Egle Maximowitsch (link), in which such CT states have been modeled, but to the best of our knowledge, these results have not yet been shared with the community in a peer-reviewed paper. Nevertheless, because CT states in general have much smaller oscillator strengths than the π-π* excitations, we assume that the initial excited state is the localized π-π* excited state of the chromophore and not a CT state involving an electron transfer between the His260 sidechain and the chromophore. To the best or our knowledge, there is also no experimental evidence for that. Nevertheless, to alert the reader to the possibility that CT states have been suggested to play a role in the photo-activation process, we provide a reference to the PhD thesis.

Page 4 SI: ”We note that because the His260 residue, which interacts with the C-ring of the chromophore via π-stacking, was not included in the QM region, we could not investigate a recent hypothesis that a charge-transfer (CT) excitation between this residue and the chromophore plays a role in the photo-activation mechanism.[11] However, because these CT states have a significantly smaller oscillator strength than the main π-π* transition, we consider it a valid approximation to focus exclusively on the π-π* S_1 state in this work.”
